# HIV serostatus disclosure to sexual partner: a survey among women in Tehran, Iran

**DOI:** 10.1186/s40001-022-00663-6

**Published:** 2022-04-08

**Authors:** Zahra Pashaei, Fatemeh Oskouie, Maziar Moradi-Lakeh, Shayesteh Jahanfar, Shima Haghani

**Affiliations:** 1 Nursing Care Research Center, Iran University of Medical Sciences, Tehran, Iran; 2 Department of Community Health Nursing, School of Nursing and Midwifery, Iran University of Medical Sciences, Tehran, Iran; 3 Iranian Research Center for HIV/AIDS (IRCHA), Iran Nursing Care, Tehran University of Medical Sciences, Tehran, Iran; 4 Preventive Medicine and Public Health Research Center, Psychosocial Health Research Institute, Department of Community Medicine, School of Medicine, Iran University of Medical Sciences, Tehran, Iran; 5 MPH Program, Department of Public Health and Community Medicine, Tufts University School of Medicine, Boston, USA

**Keywords:** HIV, Disclosure, Women, Reflection

## Abstract

**Background:**

Disclosure of HIV-positive status in women is associated with many factors. Consequently, status disclosure remains a challenge for Iranian women living with HIV. This study aimed to assess the prevalence, related factors, and reflections of HIV-positive status disclosure to a sexual partner(s) among Iranian women living with HIV.

**Methods:**

A cross-sectional study was conducted on 170 HIV-seropositive women. Participants were selected from patients registered in the largest HIV clinic and HIV-positive club of Iran. The “HIV disclosure” questionnaire had 38-items and all the interviews were administered by the researcher. Data were analyzed using SPSS version 21.0 software. We used a logistic regression method to calculate the crude odds ratio (COR) and the adjusted odds ratio (AOR) for self-disclosure as the independent predictor variable and the dependent variable, respectively.

**Results:**

One hundred and seventy HIV-positive women were enrolled. Most of them had disclosed their HIV status to at least one person (94.1%) and their sexual partners (86.5%). In the univariate analysis, being married (COR = 18.66, 95% CI 5.63–61.87), living with a sexual partner (COR = 4.72, 95% CI 1.92–11.62), being aware of sexual partners’ HIV status (COR = 6.20, 95% CI 1.79–21.49), and gaining the support of sexual partner (COR = 9.08, 95% CI 3.48–23.64) were associated with higher odds of HIV status disclosure. In the multivariate analysis, being aware of sexual partners’ HIV status, and gaining the support of sexual partners remained associated with HIV status disclosure. Most women reported a positive reflection from their sexual partners after disclosure, however, negative reflections from society were more common compared to sexual partners and family members.

**Conclusion:**

This study shows high overall HIV disclosure proportions. It should be noted that a large number of women were infected by their sexual partners, especially by their spouses. The high rate of transmission in married people indicates an urgent need for more emphasis on appropriate prevention behaviors by infected partners.

## Background

Newly reported HIV diagnosis rates in Iran have been steadily increasing [1]. Iran is one of the countries that follows the latest HIV strategy of the WHO by raising awareness about HIV/AIDS, identifying newly infected individuals with HIV testing in high-risk populations, and virally suppressing people living with HIV without considering CD4 count [2, 3]. Based on the official reports the most common mode of HIV transmission in Iran is changing from injectable addiction to high-risk sexual transmission, as it has been increased to 40% of all modes of transmission [4].

Disclosure of HIV status to sexual partners has essential implications for adherence to treatment, the health of people living with HIV (PLHIV), and preventing new HIV infections [5]. Besides, it can increase voluntary HIV counseling, testing, and safer sex behaviors [6, 7]. The rate of disclosure in women living with HIV (WLHIV) to sexual partners varies widely according to countries and their cultures [8, 9]. Previous studies found that women are less inclined to disclose their positive status to their sexual partners than men in Iran [10] which is in contrast to other countries [6, 11]. The critical barriers to status disclosure in WLHIV are often cited as stigma, loss of children [10], fear of abandonment, loss of economic support, discrimination, violence, and shame [12].

HIV-positive disclosure is associated with severe implications. Furthermore, gender inequalities make women more vulnerable to HIV-related stigma. According to gender norms, men have control over women, and as a result, women have less autonomy in deciding whether to have children, engage in sexual activity, or receive health care [13]. Unfortunately, married women are supposed to have continuous negotiation about the use of condoms since most people believe that there is no need for safe sex in marriage [13, 14]. Studies show that globally 30% of women experience physical and sexual violence by an intimate partner in their lifetime [15, 16] and intimate partner violence increases the risk of sexually transmitted infections [15]. As a result, the fear of being exposed to violence is one of the main obstacles to HIV disclosure [17]. Some studies which assessed reflections of HIV disclosure in WLHIV indicated violence as a negative reflection in various ranges [18, 19].

One of the main barriers to disclosure is the fear of losing financial support and food insecurity. Women’s limited access to employment and less property makes them vulnerable to food insecurity and its consequences [20]. For instance, WLHIV who experience food insecurity is more likely to report transactional sex and start a new relationship to have financial support [21]. Additionally, food insecurity among women is associated with significantly higher odds of inconsistent condom use with a non-primary partner, and transactional sex [22]. Hence, the economic dependency of women and their fear of abandonment are among the main reasons for the non-disclosure of HIV infection [17, 23].

Stigma remains a crucial issue for those living with HIV and it is experienced by WLHIV in a variety of levels, such as family, community, or health care settings [24, 25]. Due to health routines like pre-pregnancy tests in most settings, women are tested first for HIV and subsequently blamed for bringing HIV infection to the family, as well as adultery [23, 26]. Some studies have shown that HIV-related stigma has led to inequities in gender and sexualities [27]. Women prefer to disclose their HIV status to limited persons, as a result, they face serious health problems including less access to treatment or supports of HIV-related health services and compromising adherence to antiretroviral therapy (ART) [28].

According to the U=U (undetectable equals untransmittable) campaign started from a study conducted in May 2011, early onset of antiretroviral therapy is associated with a much lower risk of a linked partner infection as compared to delayed ART [29, 30]. People living with HIV (PLHIV) who have undetectable viral load do not transmit HIV infection to their partners [31]. Therefore, starting ART in all PLHIV regardless of the CD4 + count is beneficial in preventing infection transmission to a sexual partner [32]. This study aimed to describe the proportions, barriers, and reflections of HIV-positive status disclosure to the sexual partner(s) in HIV-infected women referred to the Positive Club and Consulting Clinic of Behavioral‏ Disorders of Imam Khomeini Hospital in Tehran.

## Methods

### Study design and setting

This cross-sectional study was conducted in the Consulting Clinic of Behavioral‏ Disorders and Positive Club of Imam Khomeini Hospital in Tehran, Iran. At the time of the study, both sites were implementing the World Health Organization (WHO) option B regimen per national guidelines including initiation of ART regardless of CD4 count [3]. The Consulting Clinic of Behavioral‏ Disorders of Imam Khomeini Hospital is one of the largest centers‏ that provide services for people living with HIV/AIDS and makes it‏ possible for patients to use specialized medical assistance as it is‏ part of a major academic hospital. This study was designed to understand the rates and context of disclosure (or lack thereof) among women living with HIV. Female participants were included in this study to explore disclosure status to sexual partners, reasons for disclosure or non-disclosure, and sexual partners’ reflections. The frequency of disclosure to other individuals and their reflections were also examined.

### Study participants and recruitment

We invited every women attending the centers to participate in an interview. A total number of 192 WLHIV accepted the invitation, out of which 170 WLHIV met the criteria of the study and fully answered the questions. These participants must have met the following inclusion criteria; an HIV-positive woman who was at least 18 years of age, ever had a spouse or casual sexual partner, was aware of sero-positivity for more than 6 months, had an Iranian origin, and was receiving or had previously received health services at the study sites. Informed written consent was obtained and adequate cooperation was provided to them in filling out the questionnaire. In our study, status disclosure is defined as the previous talk about HIV test results with sexual partners, relatives, and friends. The frequency of disclosure, related factors, and reflection of the sexual partner (s) and others to disclosure were examined.

### Data collection and analyses

#### Data collection

Face-to-face interviews were conducted by two trained researchers using a semi-structured questionnaire in a private room. The interviewer identified eligible women according to criteria. Before starting the interview, informed consent was obtained. A six-section “HIV disclosure” questionnaire was used to collect quantitative data. The main questionnaire was designed by Teklemariam Gultie’s research team [33] and its validity and reliability have been confirmed by its designers. It consists of 5 sections, and 57 items including socio-demographic characteristics of participants and sexual partners, health and healthcare factors, disclosure status, and reasons for disclosure/non-disclosure. A sixth section has been added in our study to assess the reflections of disclosure to others.

Furthermore, the main questionnaire was in English so we had to translate it into the Persian language. Two native translators who were experienced in medical literature did the translation from English to Persian. Then the primary translation was reviewed for readability and understandability. Afterward, two other bilingual and experienced translators who knew about the aim of the study translated the questionnaire back into English. The obtained version of the questionnaire was sent to the designer to confirm that the meaning of the questions had not changed. To determine the validity of the questionnaire, the Persian version was assessed by 13 HIV expert researchers. After evaluating the questionnaire some questions turned out to be consonant with Iranian culture while some others were removed because they did not meet the study goals. The validity of the reformed questionnaire was measured by answering the questions by 30 participants twice in 10 days. In addition, a final section was added to the questionnaire to assess reflections on disclosure. The first section of the reformed questionnaire collected information about participant socio-demographics (including gender, age, marital status, income, occupation, and educational level). The second section gathered partners’ characteristics (including education, living together, HIV status, and the number of partners). The third section collected health and healthcare-related factors for disclosing to sexual partners (including how long the person had HIV, who initiated to test, counseling, antiretroviral therapy). The fourth section collected disclosure status and sexual experiences of participants (including the importance of disclosure, disclosed to whom, disclosure status, perceived factors to reveal, unprotected sex, support group). The critical question of this section posed to participants was “After you knew that you were HIV-positive, to whom did you disclose?”. This question was followed by other questions to determine the process and reasons for disclosure or non-disclosure to sexual partners in the fifth section (non-disclosing factors only for a non-disclosed individual). The sixth extra section collected reflections of revelation in those who disclosed to at least one person, by asking “What happened when you told another person(s) about your HIV status?”.

#### Data analysis

The collected data were entered and analyzed using SPSS version 21.0 (IBM Corp., Armonk, NY, USA) for Windows. To describe the variables, descriptive statistics were used and bivariate analyses and multivariate logistic regressions were used to investigate associations between disclosure of HIV status, socio-demographic characteristics of participants and their sexual partners, healthcare-related factors, and their relationship with the outcome variable. We used the binary logistic regression method to calculate the crude odds ratio (COR) of all variables as independent predictor variables. Then a multivariate model was conducted to calculate the adjusted odds ratio (AOR) for all significant variables in bivariate analyses as dependent variables. Odds ratio with 95% CI (confidence interval) at *p* < 0.05 was used to determine the significant level of association between predictors and disclosure.

## Results

### Characteristics of participants

During the data collection stage, 192 women were interviewed who matched the study criteria. Twenty-two of them (11.4%) did not completely fill the questionnaire and were excluded from the study which led to a study population of 170 patients. More than half of them (55.3%) were 40 years old or older. The mean age of the participants was 41.59 ± 8.7 years and about three-quarters of them (*n* = 127; 74.7%) were married. Unemployment was very common (75.3%), and the majority of the women (*n* = 111; 65.3%) had no monthly income (Table 1). Most of the women (*n* = 124; 77.5%) were married and never had a casual partner(s) while a few of them (*n* = 41; 24.1%) have had two or more sexual partners. The majority of the women (*n* = 143; 84.1%) reported to be infected by their spouses (*n* = 116; 68.2%) or their casual partners (*n* = 27, 15.9%). Nonetheless, in a small number of them (*n* = 42; 24.7%) the sexual partner had asked them to take the HIV test. Women who were reported to be infected by a sexual partner were inferred through participants’ self-declaration. Out of the 143 women who reported to be infected by sexual partners, 106 of them (91.3%) live together with their sexual partners and 34 (29.3%) of them have children at home. Also, the rest of the women (*n* = 27) who reported being infected through other ways than their sexual partners, 15 (55.5%) live with their sexual partners with 7 (26%) of them having children at home. Fortunately, most of the study participants (92.9%) were on antiretroviral therapy.

**Table 1:** Socio-demographic characteristics of respondents

	*n* (%)
Age (years)	
≤ 29	15 (8.8)
30–39	61 (35.9)
40–49	64 (37.6)
50–59	24 (14.1)
> 60	6 (3.5)
Duration of HIV (years)	
< 1	12 (7.1)
1–5	60 (42.8)
6–10	67 (47.8)
> 10	31 (18.2)
Monthly income	
No income	111 (65.3)
≤ 250 dollar	28 (16.5)
> 250 dollar	13 (18.2)
Occupation	
Unemployed	128 (75.3)
Employed	40 (23.5)
Education	
Illiterate	15 (8.8)
Primary school (years 1–5)	19 (11.2)
Primary school (years 6–9)	27 (15.9)
Secondary school (years 10–12)	48 (28.2)
Diploma	37 (21.8)
Academic education	24 (14.1)
Marital status	
Married	127 (74.7)
Unmarried	15 (8.8)
Divorced	15 (8.8)
Widow	13 (7.6)

In cases of data missing, the total percentage did not reach 100

### Disclosure proportions

Among the 170 subjects, disclosure of HIV-positive status to at least one person occurred for 160 (94.1%), out of which 145 (85.3%) had disclosed to their spouses or casual partners. Ten women (5.9%) had not disclosed their HIV status to anyone (Table 2). More than half of the women (*n* = 92; 54.1%) had previously discussed their HIV status with their sexual partners before doing the test. About 33 percent (*n* = 57) of WLHIV had disclosed immediately, 47 (27.6%) had disclosed in less than 2 months after diagnosis, and 51 (30%) only disclosed their status after 2 months of diagnosis. There were 128 (75.3%) women who had HIV-positive partners while 21 participants (12.3%) reported having HIV-negative partners and 21 of them did not answer the question of the HIV status of their sexual partners. Approximately 19% (*n* = 32) of the women were members of the Positive Club and 23 of them stated that the advice of HIV healthcare facilities staff had encouraged them to disclose their seropositive status. Most of the participants (*n* = 156; 91.7%) stated that HIV status disclosure to sexual partners is essential.

**Table 2:** HIV status disclosure, disclosure to whom, reasons for disclosure, and non-disclosure to a sexual partner

	*n* (%)
HIV status disclosed	
Yes	160 (94.1)
No	10 (5.9)
Disclosed to (*n* = 160)	
Spouse	144 (90)
Casual partners	3 (1.8)
Parents	58 (36.2)
Children	8 (5)
Friends	13 (8.1)
Reason for disclosure (*n* = 145)	
Gain support from a sexual partner	120 (83.8)
Decrease transmission of HIV	30 (20.6)
Benefit the sexual partner to get medical care	8 (5.5)
Adherent to ARV drug therapy	27 (18.6)
Presence of ARV drugs in the home	14 (9.6)
Spiritual responsibility	16 (11)
Reason for non-disclosure (*n* = 25)	
Fear of abandonment	19 (76)
Fear of shaming	7 (28)
Fear of confidentiality	12 (48)
Sexual partner died	1 (4)
He might hurt me physically	5 (20)
Currently not together	4 (16)
Fear of accusation of infidelity	5 (20)
Fear of stigma and discrimination	7 (28)
I do not want him to worry	1 (4)

In cases of data missing, the total percentage did not reach 100

### Barriers to disclosure

Among the 23 individuals who did not disclose to their sexual partners, the main reason for non-disclosure was the fear of abandonment (*n* = 19; 82.6%). It was closely tied to the fear of confidentiality and trust in sexual partners (*n* = 12; 48%). Moreover, they prefer to keep their status secret because they do not have trust in their partners in keeping it confidential and about two-thirds of them (*n* = 7; 28%) cited shame and the fear of stigma and discrimination as other important reasons (Table 2). Three of the non-disclosed women had intended to disclose seropositive status at least once but later changed their minds.

### Factors associated with disclosure of HIV status to a sexual partner

The main reason for disclosing HIV status was to gain the support of sexual partners (*n* = 120; 83.8%) (Table 2). Another reason was initiating antiretroviral therapy (*n* = 54; 37.2%). Talking to an HIV-positive person(s) (*n* = 28; 16.5%) and the effects of public media (*n* = 15; 8.8%) were mentioned as effective factors for deciding to disclose their status. Women who were married had an 18-fold increase in odds of HIV serostatus disclosure to partners than those who were single (COR = 18.66, 95% CI 5.63–61.87) (Table 3). Living with a sexual partner positively influenced disclosure to a sexual partner (COR = 4.72, 95% CI 1.92–11.62). Also, disclosure to gain support from a sexual partner (COR = 9.08, 95% CI 3.48–23.64) was positively associated with HIV-seropositive status disclosure to a sexual partner(s). Those who knew the sexual partner’s HIV status were 6 times more likely to disclose their HIV serostatus to sexual partners compared to individuals who were not aware of the partner’s status (COR = 6.20, 95% CI 1.79–21.49). Habitant status, age, educational status, employment status, income, how to become infected, duration of knowing about HIV status, partners’ HIV status, with who to go for a test, prior discussion about HIV, ART status, and being a member of the Positive Club were not associated with HIV status disclosure. The association of socio-demographic and healthcare factors of the participants with the disclosure is presented in Table 4.

**Table 3:** Disclosure dispersion in who disclosed to at least one person (*n* = 160)

Disclosure	*n* (%)
Only to a sexual partner	79 (49.3)
To sexual partner and one other person	53 (33.1)
To sexual partner and two other people or more	13 (8.1)
Not to a sexual partner and at least to one person	15 (9.3)

**Table 4:** Association of demographic, socioeconomic, health, and health care factors to the disclosure of HIV status

Variables	Disclosure status		Disclosed HIV to sexual partner	
	Yes *n*(%)	No *n*(%)	Cr OR (95% CI) *p	Adj. OR (95% CI) *p
Residence				
Urban	140 (82.3)	25 (14.7)	2.43 (0.44–13.30) *p* = 0.30	
Rural	7 (4.1)	2 (1.1)		
Age, years				
< 40	63 (37)	13 (7.6)	1.41 (0.60–3.30) *p* = 0.42	
≥ 40	82 (48.2)	12 (7)		
Marital status				
Single	5 (2.9)	10 (5.8)	18.66 (5.63–61.87) *p < 0.001	3.64 (0.65–17.49) *p* = 0.72
Married/divorced/widowed	140 (82.3)	15 (8.8)		
Education status				
Undergraduate	94 (55.2)	15 (8.8)	0.81 (0.34–1.94) *p* = 0.64	
Graduate and above	51 (30)	10 (5.8)		
Living status				
With sexual partner	116 (68.2)	11 (6.4)	4.72 (1.92–11.62) *p = 0.001	1.87 (0.82–4.11) *p* = 0.82
With others	29 (17)	14 (8.2)		
Employment				
Unemployed	111 (65.2)	17 (10)	0.97 (0.35–2.62) *p* = 0.95	
Employed	34 (20)	6 (3.5)		
Income				
None	98 (57.6)	13 (7.6)	1.51(0.55–4.17) *p* = 0.42	
< 250 dollar	22 (12.9)	6 (3.5)		
> 250 dollar	13 (7.6)	5 (2.9)		
Duration of knowing about HIV status				
≤ 1 year	11 (6.5)	1 (0.6)	0.81 (0.62–1.19) *p* = 0.62	
2–6 years	76 (44.7)	4 (2.3)		
7–10 years	45 (26.4)	2 (1.2)		
> 10 years	28 (16.5)	3 (1.8)		
How infected to HIV				
Sexual partner	121 (71.1)	22 (12.9)	0.68 (0.19–2.48) *p* = 0.56	
Blood transfusion/injectable addiction/illegal abortion/hospital	24(14.1)	3 (1.7)		
Knowing sexual partners’ HIV status				
Yes	134 (78.8)	12 (7)	6.20 (1.79–21.49) *p = 0.04	3.89 (0.82–16.34) *p = 0.02
No	9 (5.2)	4 (2.3)		
Partners’ HIV status				
Positive	120 (72.9)	8 (2.3)	3.52 (0.95–12.99) *p* = 0.05	
Negative	17 (11.1)	4 (1.1)		
With whom to go to test				
Alone	125 (73.5)	18 (10.5)	0.41 (0.15–1.11) *p* = 0.07	
With spouse/casual partners/parent/relatives/peer group	20 (11.7)	7 (4.1)		
Prior discussion				
Yes	85 (50)	12 (7)	1.13 (0.43–2.96) *p* = 0.79	
No	50 (29.4)	8 (4.7)		
ART status				
Pre-ART	8 (4.7)	3 (1.7)	2.31 (0.57–9.41) *p* = 0.24	
ART	136 (80)	22 (12.9)		
Being a member of positive club				
Yes	26 (15.2)	6 (3.5)	0.69 (0.25–1.90) *p* = 0.47	
No	119 (70)	16 (9.4)		
Disclose to get support from sexual partner (disclosed individuals *n* = 145)				
Yes	113 (77.9)		9.08 (3.48–23.64) *p < 0.001	4.21 (0.51–5.68) *p = 0.004
No	32 (22.1)			

In cases of data missing, the total percentage did not reach 100

*Cr OR* crude odds ratio, *OR* adjusted odds ratio

*95% significant at *p*-value < 0.05

In the multivariate analysis adjusted for all variables associated with HIV status disclosure in the univariate analysis, HIV status disclosure remained significantly associated with being aware of sexual partners’ HIV status (AOR = 3.89, 95% CI 0.82–16.34) as well as the disclosure to gain support from sexual partners (AOR = 4.21, 95% CI 0.51–5.68) (Table 4).

### Reflections to HIV status disclosure

The majority of women who disclosed to their sexual partner(s) (*n* = 145) reported positive reflections from their sexual partners (*n* = 119; 82%), i.e., they confirmed that their partners were supportive and understanding and did not show violence or bad behavior. About one in four women (*n* = 40; 25%) stated that disclosure encouraged sexual partners to take HIV tests though a smaller proportion (*n* = 15; 9.3%) had experienced divorce or separation after disclosure and 14 of them (8.7%) were abandoned by their sexual partners (Table 5).

**Table 5:** Reflection to HIV status disclosure to at least one person (*n* = 160)

Reflections	*n* (%)
Sexual partner decided to do a test	40 (25)
Divorce/separate from a sexual partner	15 (9.3)
Abandoned by a sexual partner	14 (8.7)
Support from a sexual partner	119 (74.3)
Negative reactions of society	47 (29.3)
Ignorance by society	29 (18.1)
Stigma and discrimination	83 (51.8)
Support from family	66 (41.2)

In those who disclosed to at least one person (including sexual partner or anyone else) (*n* = 160), more than half (*n* = 83; 51.8%) declared that they experienced stigmatization and discrimination after the disclosure. Similarly, some of them experienced negative reflections from the society (*n* = 47; 29.3%) and ignorance by the community (*n* = 29; 18.1%). Despite this, more than one-third of them (*n* = 66; 41.2%) reported that they received support from their families (Table 5).

## Discussion

The analysis of this study shows that the prevalence of status disclosure to partners among HIV-positive women who were older than 18 years is 85.2%. The findings of this study are similar to disclosure rates in studies carried out in Uganda and Kenya reporting 85.4% and 80% HIV-status disclosure rates by women to their male partners, respectively [34, 35]. Conversely, the prevalence observed in this study is higher compared to previous studies conducted in Uganda (57%) and Tanzania (66%) [34, 36]. Additionally, our study found out that the rate of disclosure to at least one person (including a sexual partner or anyone else) was high (94.1%). Other studies also found similar or higher rates of disclosure to at least one person [37, 38]. Among those who disclosed to at least one person, nearly half of them (49.3%) only disclosed to their sexual partners and only 8.1% disclosed to two or more people apart from their sexual partners.

Unfortunately, 25 women (14%) who had sexual partners were married or cohabiting but failed to disclose their status to their partners. Furthermore, 8 of them claim that after being diagnosed with HIV they have had sex without a condom which increases the likelihood of transmitting the infection to their sexual partners. One of the main reasons identified for non-disclosure was stigmatization. Half of the women perceive stigma from a sexual partner, family, and community as well as ignorance and negative reactions from society, especially in healthcare centers. These findings are similar to findings of two studies conducted in Iran which claimed that HIV-related stigma leads to HIV status non-disclosure [39, 40]. Some studies reported that in some settings, healthcare providers discourage WLHIV from having sex and planning to have children [41, 42], blaming them for transmitting the infection [26]. A study conducted in Kenya to assess social concerns related to HIV status disclosure showed that isolation or lack of support from family or friends, fear of separation, and fear of conflict with a partner were the concerns of women which led to non-disclosure of their status to their sexual partners. Therefore, challenging stigmatization should be considered as a basic pillar to empower WLHIV and enhance coping with new conditions after infection in HIV-related health services in Iran [35]. Every single woman should practice overcoming internal stigma, and HIV disclosure-related concerns in HIV support groups so that she could fight for equal rights in the community against stigmatization. Subsequently, she could be able to disclose her status with the least delay as well as gain the support of both her family and sexual partner to deal with the potential difficulties of being diagnosed with HIV. Ultimately, HIV program staff have a crucial role in encouraging WLHIV to cope with stigma, manage their anxiety and pre-disclose their concerns.

In our study, most of the women were infected by their sexual partners accounting for 84.1% of all modes of transmission. This finding is consistent with recent changes in the main route of HIV infection transmission in Iran. Multiple factors such as cultural, economic, and social factors combined with transactional sex, lack of comprehensive sexual and reproductive health services including HIV testing, and minimal access to treatment have led to this change. The shift in the epidemiological pattern of the disease in recent years could seriously threaten the health and safety of the Iranian community, especially women [4]. A previous study conducted in Iran strongly supports this phenomenon by reporting sexual relationships as the route of transmission in most participants [43]. Among women infected by their sexual partners, a significant number (81.1%) were affected by their spouses. This may be attributed to the pressure married women face from men's power and domestic violence to have unprotected sex though gender norms expect couples to have consensual sex [44]. Furthermore, the rate of condom use in developing countries including Iran remains low [15, 45]. Domestic violence against women in Iran was reported to be 66% from a meta-analysis conducted by Hajnasiri et al. [46]. The stigmatization and criminalization of HIV could be more in women living with HIV [47] Moreover, women would be blamed for bringing HIV into their families if they are tested positively first [17, 23].

Out of the women who were infected by their sexual partners, 91.3% continued to live with them. This could be linked to the limited access for women to formal jobs, consequently, they do not have (or have low) income and are unable to afford living expenses. A large number of participants in this study (65.3%) had no income. Women are usually dominated by men due to inadequate income, and men are seen as the heads of their families hence, have control over every marital, economic, and social affair of life [48]. Financial empowerment through increasing self-esteem could enable women to be more involved in the household, sexual, and reproductive decision-making thereby decreasing gender inequalities [49, 50]. Furthermore, sero-positivity status disclosure to sexual partners has a positive impact on women who have high incomes and have no dependency on their sexual partner to pay for food, rent, and other fees [8]. Another reason for the high rate of inhabitation of participants with their sexual partners may be due to the complicated government laws for these cases in Iran as there is no HIV-related law to let infected women seek a divorce. Thus, a social support system is needed to support women to help them survive after divorce by decreasing the risk of poverty and enable them to raise their children without impaired cognitive development and food insecurity.

Interestingly, a great number of participants believed that HIV disclosure to sexual partners is a spiritual responsibility, revealing that it may decrease the chance of transmission to a sexual partner. This further allows them to get tested for HIV and start HIV-related care early if needed. More also, there is an association between being married and HIV-positive status disclosure to a partner from our study. Participants who were married were more likely to disclose their HIV serostatus to their sexual partners than others. In concordance, a study by Damian et al. supports this finding as they reported that disclosure is higher among women who are married compared to single women [36]. Furthermore, women who live in the same house with their partners are 4.7 times more likely to disclose their status to their partners than those who do not as similarly reported by Deribe et al. and Skunodom et al*.* [11, 51]. High disclosure rates in those who live with their sexual partner may be due to the close ties between them, especially those who perceive their relationship as supportive, loving, and trusting would disclose without fear of rejection or abandonment [52].

In the multivariate analysis, being aware of a partner’s HIV status was found to be an independent factor associated with disclosure to the partner. Additionally, the study participants who knew their partner’s HIV status were 4.9 times more likely to disclose their HIV-positive results to sexual partners compared to those who were unaware of the sexual partner’s HIV status. Similar findings from Tanzania and London were reported in two studies indicating the higher tendency of women to disclose their HIV status to their partners when they know about their partner’s HIV status [53, 54]. Moreover, women who disclose their status to gain sexual partner support are 9 times more likely to disclose than other women as seen in our analysis. In the multivariate analysis, disclosure to gain the support of a sexual partner was found to be an independent factor associated with disclosure to a sexual partner. The support of sexual partners includes understanding, respect, and psychological support which is one of the main concerns of women before the disclosure [35].

Another aspect of this study shows that most of the women who participated in this study reported positive reflections from their sexual partner(s) after disclosure (82%) and 25% of them decided to take the HIV test. The support from the sexual partner was described as understanding and without violence and bad behavior. In similar studies done in Tanzania and Kenya, participants reported 91.7% and 87.5% positive reflections, respectively [54, 55]. Due to this increasing trend, it is advised that HIV care and counseling centers should encourage and counsel people living with HIV to disclose their status [18, 56]. However, some women reported negative reactions including divorce/separation, abandonment, stigma, and discrimination from their partners [11, 35]. For those who disclosed their status to at least one person (including sexual partner or anyone else) (*n* = 160), almost all of the reflections received from the community were negative such as stigmatization and discrimination, ignorance, and encounter with improper behavior in society even as far as healthcare centers.

The present study documents that the prevalence of disclosure among WLHIV is high though it may not be expandable to the whole HIV-positive population in Iran. Additionally, it shows that being aware of a sexual partner’s HIV serostatus and gaining support from a sexual partner are significantly associated with HIV status disclosure. Although more research is needed to elucidate the exact effect of gender inequality in the disclosure of HIV status, it could be postulated as one of the critical factors that influence WLHIV’s life. A limitation of this study arises from the population, which was drawn from only the Consulting Clinic of Behavioral‏ Disorders and Positive Club of Imam Khomeini Hospital. Hence, the HIV status disclosure rate may be different from other People Living with HIV who do not seek care or live in rural areas since the status of patients attending hospitals is known to their healthcare providers. Thus, they may feel comfortable disclosing their seropositive status to others as well. Also, the number of people who did not disclose to anyone (except healthcare providers) was less, therefore we could not perform the statistical test for them and subsequently reported non-disclosure reasons in percentages.

## Conclusion

The findings of this study show high overall HIV disclosure proportions. It should be noted that a large number of women were infected by their sexual partners, especially by their spouses. The high rate of transmission in married people indicates an urgent need for more emphasis on appropriate prevention behaviors by infected partners. Additionally, it is well established that the prevention programs and early testing programs reduce the probability of infection transmission, especially in couples, and have significant economic and social benefits. Also, further studies are needed to make a reliable conclusion on the deterrent reasons of individuals from disclosing HIV status to sexual partners. The involvement of partners of PLHIV can create a sense of companionship and enable PLHIV to regain control over their lives. Information about sexual behavior is essential to plan for preventive strategy and to correct public perception of sexual behavior hence, more studies are needed about sexual behavior in Asia and the Middle East including Iran. Lastly, targeted training of healthcare providers to combat stigma in their facilities could guarantee the correct provision of information about family planning, HIV, and other STIs to patients. To implement U=U successfully and significantly reduce infection transmission among sexual partners, continuous viral monitoring to a level of < 200 copies/ml, easy access to testing and treatment as well as interventions to attain and sustain viral suppression are needed.

## Data Availability

The data that support the findings of this study are available from the corresponding author, [FO], upon reasonable request.

## Abbreviations

AIDS: Acquired immunodeficiency syndrome
AOR: Adjusted odds ratio
ART: Antiretroviral therapy
ARV: Antiretroviral
COR: Crude odds ratio
HIV: Human immunodeficiency virus
PLHIV: People living with HIV
U=U: Undetectable equals untransmittable
WHO: World Health Organization
WLHIV: Women living with HIV
